# Buildings Contemplated

**Published:** 1913-10-25

**Authors:** 


### Buildings Contemplated.

Bierley.—The Health Committee has resolved to recommend the Bradford Corporation to enlarge the Bierley Hall Sanatorium.

Blyth.—The Urban District Council has resolved to provide a new hospital.

City of Liverpool Sanatorium.—Thirty designs were submitted in open competition by architects in response to the invitation by the Port Sanitary and Hospitals Committee of Liverpool for a sanatorium at Fazakerley. Accommodation for 250 beds is required. The result of the competition is now announced, and the selected design is by Messrs. Hooper, of Redhill, Surrey; the second premium goes to Messrs. Brooke and Elcock, of Manchester; and the third to Mr. T. W. Haigh, of Liverpool. The plans submitted are on exhibition in the large lecture hall of the Central Technical School, Byrom Street, Liverpool, till the 25th inst.

Grays.—An agreement has been entered into between the Essex County Council and the Orsett Joint Hospital Board for the erection and furnishing by the County Council of a pavilion at the Grays Hospital for eight tuberculosis patients.

Hawkmoor.—Plans have been prepared for a sanatorium for the County of Devon at Hawkmoor. The estimated cost of providing four pavilions and a dining-hall is £12,370.

Odsal.—The Health Committee of the Bradford Corporation has approved plans for a temporary small-pox hospital which is estimated to cost £3,600.

Pennington.—The Local Government Board has approved plans submitted by the Lancashire County Council for the erection of a sanatorium at Pennington, and tenders for the work will shortly be invited. Messrs. Settle and Brundritt are the architects. The cost, including the site, is estimated at £16,000.

Sandon.—The Essex County Council has purchased Spereham's Farm, with ninety-eight acres of land, as a site for a sanatorium, at a cost of £1,800.

Wrexham.—The Wrexham Rural District Council has under consideration a proposal for building a small-pox hospital at an estimated cost of £1,490.

